# Face Cooling During Swimming Training in Tropical Condition

**DOI:** 10.3389/fpsyg.2021.622184

**Published:** 2021-04-23

**Authors:** Florence Riera, Roland Monjo, Guillaume R. Coudevylle, Henri Meric, Olivier Hue

**Affiliations:** ^1^Laboratory ACTES, UPRES-EA 3596, University of the French West Indies, Point-à-Pitre, France; ^2^Laboratory IMAGE, UMR ESPACE DEV 228, University of Perpignan Via Domitia, Perpignan, France

**Keywords:** thermoregulation, swimming, performance, training perception, training sensation

## Abstract

The aim of this study was to test the effect of face cooling with cold water (1.2 ± 0.7^°^C) *vs.* face cooling with neutral water (28.0 ± 3.0^°^C) during high-intensity swimming training on both the core temperature (T_co_) and thermal perceptions in internationally ranked long-distance swimmers (5 men’s and 3 women’s) during 2 randomized swimming sessions. After a standardized warm-up of 1,200 m, the athletes performed a standardized training session that consisted of 2,000 m (5 × 400 m; start every 5’15”) at a best velocity then 600 m of aerobic work. Heart rate (HR) was continuously monitored during 5 × 400 m, whereas T_co_, thermal comfort (TC), and thermal sensation (TS) were measured before and after each 400 m. Before and after each 400 m, the swimmers were asked to flow 200 mL of cold water (1.2^°^C) or neutral (22^°^C) water packaged in standardized bottles on their face. The swimmers were asked don’t drink during exercise. The velocity was significantly different between cold water and neutral water (*p* < 0.004 – 71.58 m.min^–1^ ± 2.32 and 70.52 m.min^–1^ ± 1.73, respectively). The T_co_ was increased by ±0.5^°^C at race pace, under both face cooling conditions with no significant difference. No significant changes were noted in mean HR (i.e., 115 ± 9 and 114 ± 15 bpm for NW and CW, respectively). TC was higher with Cold Cooling than Neutral Cooling and TS was lower with Cold cooling compared with Neutral cooling. The changes in perceptual parameters caused by face cooling with cold water reflect the psychological impact on the physical parameters. The mean velocity was less important with face cooling whereas the heat rate and T_co_ were the same in the both conditions. The mechanism leading to these results seems to involve brain integration of signals from physiological and psychological sources.

## Introduction

Both the hot-dry and hot-wet (i.e., so-called tropical) tropical climate have been shown to decrease aerobic performance (Nielsen et al., 1993; Hue, 2011). The heat stress processes involved in this alteration are not clear, but several mechanisms have been proposed, including thermoregulatory anticipation (Schlader et al., 2010; Hue and Galy, 2012; Hue et al., 2013), and cardiovascular adjustments (Montain and Coyle, 1992), leading to decreased power output (Périard et al., 2011). A hot environment is also associated with higher thermal discomfort and a lower thermal sensation (TS; Nybo and Nielsen, 2001). This has been demonstrated for swimming (Hue and Galy, 2012; Hue et al., 2013), running (Hopkins and Hewson, 2001), and cycling (Lee and Shirreffs, 2007; Riera et al., 2016). Pre-cooling or per-cooling protocols, such as water immersion or cold air exposure, are among the strategies used to decrease the deleterious effect of the hot environment on aerobic performance. Although they may be successful, these strategies are time-consuming and logistically very difficult to apply in real sports contexts (Hue et al., 2014a,b; Trong et al., 2015; Riera et al., 2016). Much of the recent work on heat exercise tolerance is based on the assumption that body temperature plays an important role in modulating work rate or tolerance (Eijsvogels et al., 2014). However, the actual thermal afferents and their relative contribution are less clear, and the results of impaired central neuromuscular activation mediated by hyperthermia and anticipatory feedback modeling both appear to be based on the importance of the thermal state of the brain (Nelson and Nunneley, 1998).

Selective cooling of the face (FC) or head (HC) increases brain blood velocity at rest (Brown and Williams, 1982) and during exercise (Kjeld et al., 2009). Selective FC is particularly effective in the receptor for heat sensitivity to skin cooling during hyperthermal exercise (Brisson et al., 1989). Although there has been speculation that Heart rate (HR) may directly cool the brain, thereby altering hypothalamic production (Cabanac and Caputa, 1979a,b), this has been counterbalanced by more recent evidence that brain temperature is not affected, so that any consequences of head cooling appear likely to be mediated by stimulation of skin afferents (Nelson and Nunneley, 1998; Nybo et al., 2002). Armada-da-Silva et al. (2004) demonstrated that CF during a short period (14 min) of cycling (63% of maximum aerobic power) reduced hyperthermia and increased thermal comfort (TC) and RPE. Quirion et al. (1990) studied physiological and metabolic responses to facial cooling using wind during graduated maximal exercise and prolonged sub-maximal exercise for 30 min at 65% VO2 max while cycling on an ergometric bike. The results suggest that facial wind stimulation during maximal exercise does not produce stress high enough to alter metabolic and physiological responses. Palmer et al. (2001) reported a higher rate selection in runners trained in warm environments with head cooling, suggesting possible behavioral or perceptual neutralization of hyperthermia signals. This is corroborated by reports of attenuation of perceived stress and heat stress relative to physiological heat stress in fit individuals in non-compensable heat stress environments (Tikuisis et al., 2002). Cooling of the head alone has been effective in improving TC and pace selection, both before and during running in heat (Palmer et al., 2001), and the face has been identified as a site of high sudomotricity and any heat sensitivity (Cotter and Taylor, 2005). To date, all studies have focused on cooling the body or face in air and no studies have examined the effect of swimming CE on HR, speed, TC, and TS in a tropical climate. Facial cooling studies have been conducted in immersion and have been studied for exposure to cold or cold water (Riera et al., 2014; Coudevylle et al., 2019).

A particularly interesting question concerns the extent to which to how the tropical climate (i.e., hot. water in hot environment) affects the thermoregulatory processes during swimming. It is well known that the thermal balance of swimmers is regularly challenge due to the high heat transfer coefficient of water (Wade and Veghte, 1977) and it has been demonstrated that swimmers in tropical climate are impacted by the environment (Hue et al., 2007, 2013; Hue and Galy, 2012) with the increase of the thermoregulatory cost of swimming increasing the core temperature (T_co_; Hue and Galy, 2012), decreasing the performance (Hue and Galy, 2012; Hue et al., 2013), or increasing the acclimatization processes (Hue et al., 2007).

Very recently some protocols, using the absorption of very cold water during swimming, have been proposed to help cooling in internationally-ranked swimmers during events performed in tropical climate, decreasing the T_co_ or the cardiac cost of swimming (Hue et al., 2013). Because cooling the face using cold water could also be a logistically easy to apply method during open water competition event, the aim of the study was thus to determine if face-cooling (cold *vs.* neutral) water has positive results on the swim velocity, HR, T_co_, TC, and TS in swimming in hot and humid environment.

## Materials and Methods

### Subjects

Eight internationally ranked long-distance swimmers (5 men and 3 women; ranked 6–16 at the 2012 10; or 25-km European Long-Distance Swimming Championship/ranked 6–11 at the London 2012 Olympics) participated in this study. All were members of the French Team, training for 21 days in Martinique (French West Indies; mean diurnal wet bulb globe temperature at the period, WBGT: 29.6 ± 0.6^°^C; 79 ± 10% RH), and swimming twice a day (morning: 6:30 a.m.–9:00 a.m. and evening: 4:30 p.m.–7:30 p.m.) in an outdoor 50-m swimming pool (mean swimming-pool water temperature: 28.8 ± 1.2^°^C). At the time of the study, the swimmers had trained for 8 days and 119 km (i.e., 11–15 km per day) in the tropical climate. The study was approved by the Ethics Committee of the Sport Medical Centre in Guadeloupe (Ministry of Youth and Sports) and the Ethics Committee of the Training and Research in Sport Science Unit in Guadeloupe (Ministry of Higher Education and Research). All gave informed written consent, and the protocol was approved by the ethics committee of Guadeloupe University and was conducted according to the Declaration of Helsinki. In addition, this study was performed in accordance with the ethical standards of the IJSM (Harriss and Atkinson, 2014). Body mass, height and fat body mass are presented in Table 1.

**TABLE 1:** Antropometric data of subject.

**Subject**	**Age (years)**	**Height (cm)**	**Weight (Kg)**	**Fat body mass (% of body mass)**
1	29	180	76.3	14.1
2	29	184	84.3	12.5
3	34	186	81.8	15.4
4	26	176	62.8	8.1
5	26	181	74.9	16,1
6	22	172	66.8	24.7
7	24	174	68.6	23.1
8	23	174	65.8	26.2

### Experimental Design

The study took place during the usual training schedule and covered 2 swimming sessions in the evening: The swimmers performed a standardized warm-up of 1,200-m and were then asked to swim a standardized 5 × 400 m (start every 5’15”) at a best velocity then 600 m of aerobic work. Mean velocity is calculated using time to each 100 m of each 400 m bout. The swimmers were then followed for the next 2,000 m of the training schedule. After each 400 of the 5 × 400, they flew (or poured) 200 mL of water on their face at the temperature of 1.2^°^C (cold cooling) or ambient temperature of 22^°^C (neutral cooling). The use of neutral or cold face cooling was randomized for each subject over the sessions. Their internal temperature was scheduled. The trials began at the same time of day for each athlete (between 16:30 and 19:00) to check for circadian variations in T_co_ and digestion control. HR was monitored continuously during exercise using a portable telemetry unit (Suunto Memory Belt, Suunto, Vantaa, Finland) with recording every 10 s, and the data were analyzed with Suunto software. T_co_ was assessed via the gastrointestinal temperature using ingestible temperature measurement pills (CorTemp, HQ, Inc., Palmetto, FL, United States). The athletes were instructed to ingest these pills 6 to 8 h before all experimental trials to ensure the pill was out of the stomach, thereby avoiding variability in T_co_ due to pill movement or fluid/food consumption. Velocity was measured by an official timer (Omega, swiss timing, Megatek, France).

The subjects were weighed in the same conditions before and after exercise. The percentage of body fat mass was determined in these subjects. The WBGT index was monitored for the duration of the exercises (QUESTemp° 32 Portable Monitor, QUEST Technologies, Oconomowoc, WI, United States). The swimming water temperature was recorded at 1-m deep before, during and at the end of each session (YSI 409B, Yellow Springs Instruments, OH, United States).

They were asked to follow their usual diet before each session before the first session and during the experimentation duration.

### Thermal Sensation and Comfort

Whole-body (and regional, where necessary) TC and TS were determined on four (from 1, comfortable, to 4, very uncomfortable) and seven (from 1, cold to 7, hot) point scales, Gagge et al. (1967). In all experimental trials, the subject’s TC and TS were recorded by the subjects placing a vertical mark on the horizontal line (McIntyre, 1980). The corresponding level of sensation and comfort was measured at the location where these lines intersected. The subjects were fully familiarized with these scales prior to their experimental use. The familiarization included defining TC (How comfortable are you in this thermal condition?) and TS (What temperature sensation do you feel right now?; Parsons, 1993), anchoring the perceptual range, and answering the subject’s questions.

### Statistical Analysis

A Kruskal Wallis test was used to analyses impact of neutral or cold face cooling on mean velocity on 100 m, and HR; effect size has been calculated using partial eta squared. An aligned rank transforms two-way ANOVA (Mansouri et al., 2004) had been performed to analyze the impact of face cooling over time and session on TC and TS. Effect sizes were calculated using partial eta squared (η_p_^2^) and interpreted as 0.010–0.059 = small, 0.060–0.139 = medium, >0.14 = large (Cohen, 1988; Lakens, 2013). Data analysis was performed using R (r-project.org). Significance was set at 5%. Data are expressed as mean ± SD.

## Results

### Environmental Conditions

There were no significant differences in environmental conditions between sessions (i.e., the WBGT was 26.7 ± 1^°^C, 25.8 ± 0.9^°^C).

### Mean Velocity on 100 m

**FIGURE 1:**
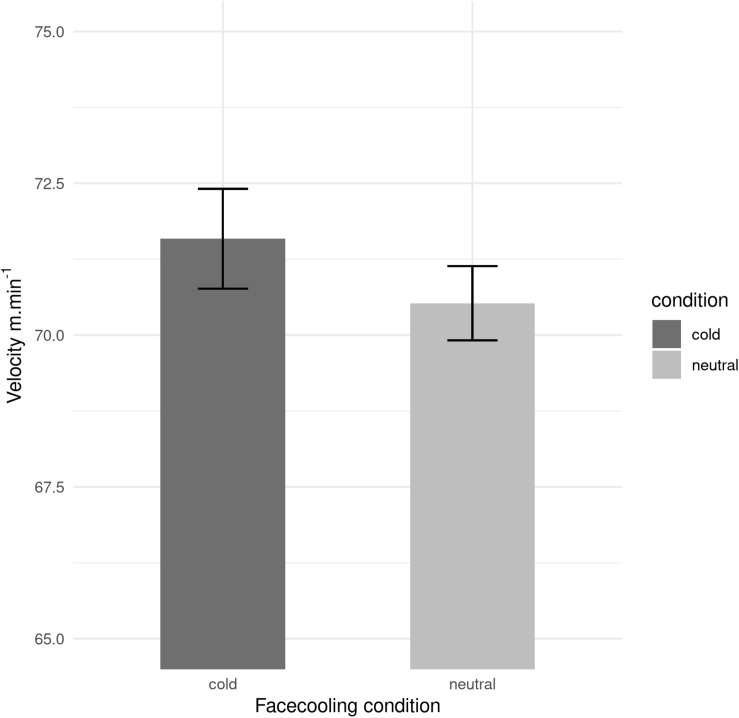
Velocity of swimmer in the both conditions: cold face cooling and neutral face cooling. Mean values and SD are shown.

There was a significantly difference between Face Cooling temperature [Neutral *vs*. Cold] and velocity (*p* < 0.004, η_p_^2^ = 0.095 moderate magnitude). The velocity is improved by the cold face cooling compared by neutral face cooling (71.58 m.min^–1^ ± 2.32 and 70.52 m.min^–1^ ± 1.73, respectively; Figure 1).

### Thermal Sensation and Thermal Comfort

**FIGURE 2:**
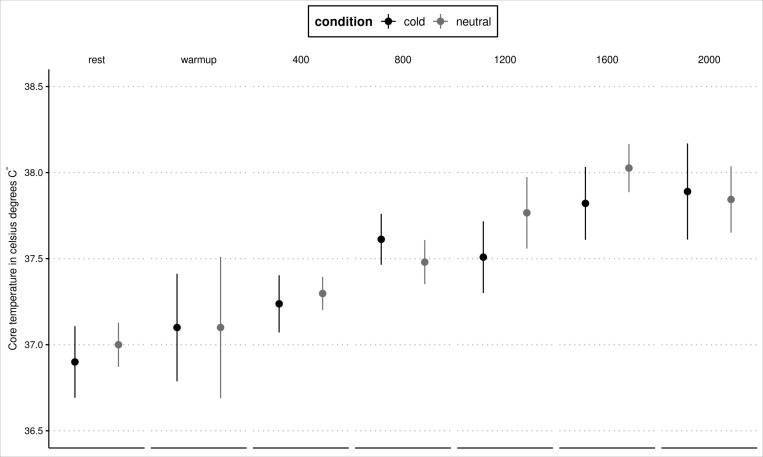
**(A)** Rating of thermal sensation and **(B)** Rating of thermal comfort at different distances with the Cold water-cooling and Neutral water cooling. Mean values and SD are shown.

Thermal Sensation was significantly affected by time (*p* < 0.02, η_p_^2^ = 0.06 moderate magnitude) and did not differ between FC temperature [Neutral *vs*. Cold] over time (*p* < 0.06). The TS was significantly improved (*p* < 0.05, η_p_^2^ = 0.011 small magnitude) between conditions at the first 400 m of 4 × 200 m. TC was significantly affected by condition and moment (*p* < 0.01, η_p_^2^ = 0.11 large magnitude). The TC is enhanced with cold cooling at 400, 800, and 1,200 m of exercise (Figure 2).

### Core Temperature and Heart Rate

**FIGURE 3:**
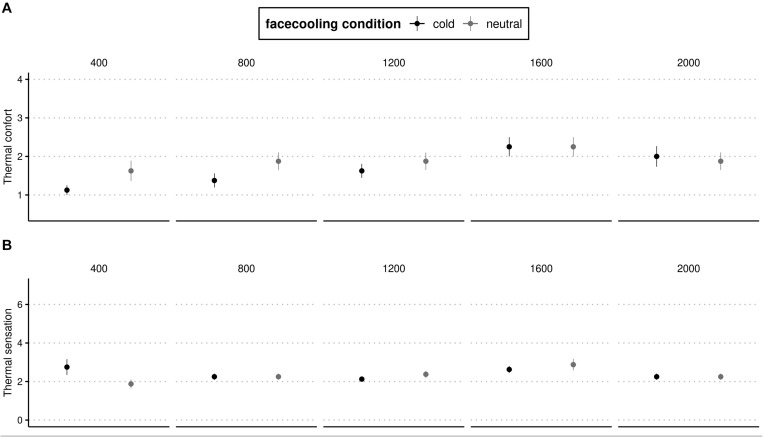
Core temperature during training with the Cold-water cooling and Neutral water-cooling. Mean values and SD are shown.

There was no significant difference in the mean T_co_ during exercise (for Neutral: 37.3 ± 0.3^°^C *vs*. Cold: 37.2 ± 0.5^°^C at the start of exercise to 37.8 ± 0.5^°^C and 37.9 ± 0.5^°^C for NC and FC, respectively - *p* > 0.05, η_p_^2^ = 0.010 small magnitude). During the sessions, T_co_ increased over time (*p* < 0.001, η_p_^2^ = 0.16 large magnitude) with no significant difference between the FC conditions (*p* = 0.19) as depicted in Figure 3.

Rest HR before training, was not significantly different over sessions (82 ± 6 bpm and 79 ± 3 bpm, for neutral cooling and cold cooling). The mean HR during training was not significantly affected by FC temperature or by the time of exercise (i.e., 115 ± 9 bpm, and 114 ± 15 bpm) for Neutral cooling and Cold cooling, respectively.

## Discussion

The aim of the study was to determine if face-cooling (cold *vs*. neutral) water has positive results on the swim velocity, HR, T_co_, TC, and TS in swimming in hot and humid environment.

Main result of our studies was that face cooling by cold water during a swimming exercise in hot water improve the mean swimming velocity of 100 m, without affected the T_co_ and HR and affected TS and TC.

### Face Cooling and Mean Swimming Velocity

In our study, we demonstrated that the mean of swimming velocity, of acclimated swimmers could also be affected in warm water by face-cooling. The swimmers improved velocity with Cold face cooling compared to Neutral face cooling (Figure 1), without significant changes in HR and T_co_ during exercise (Figure 3).

### Thermal Sensation and Thermal Comfort With Face-Cooling

Regarding psychological components, most studies focus on perceptual responses to heat stress with or without cooling intervention (Coudevylle et al., 2019). Previous studies (Nunneley et al., 1982; Armada-da-Silva et al., 2004; Cotter and Taylor, 2005; Arens et al., 2006) have demonstrated the great effect that facial cooling can have on perception/behavior responses during exercise with heat stress. Which is in agreement with our results and the fact that in our study swimming speed was slightly improved during exercise in a hot environment. But in return, the cutaneous thermal stress caused by cold water can also distract them. Swimmers feel better TC but at the same time, these cooling conditions of the face distract them from the goal of going fast. As a result, swimming speed does not improve much as swimmers lose focus from the goal of going fast (Coudevylle et al., 2019). In the end, the advantage of TC is less important than the cost generated by decreased attention. At the same time, several studies have also shown that TC is improved after acclimatization (Sunderland et al., 2008; Costa et al., 2014). It is possible that our swimmers have started acclimating to the tropical environment with several repeated exposures of 21 days during the year, which could also explain that they feel better TC during the exercise. Thus, we can conclude, according to our study, that skin temperature and associated thermal perceptions influence the voluntary choice of exercise speed (Schlader et al., 2011). Accustomed to enduring high training loads and high environmental stresses (through acclimatization), athletes perceive the difficulty of an exercise as less onerous than its objective assessment. Our results show that the cooling face temperature and thermal perceptions (sensation and comfort) are thus little able to act as modulators during exercise in water (Schlader et al., 2011).

Schlader et al. (2011) show that the effects of cooling the face during exercise in heat were manifested by a reduced perception of effort at a given intensity. To date, the only behavioral outcome related to these perceptual responses to facial cooling during heat exercise was a longer period of voluntary exhaustion. We have observed that facial thermal cooling affects the perceptual responses of the face and whole body by inhibiting heat sensation, decreasing TS and increasing TC in the first half of the exercise. However, in our model, this resulted in a longer exercise duration and therefore a higher swimming speed. Conversely, the thermal warm-up of the face increased the sensations of warmth and TC on the face and all over the body. This supports the claim that the sensitivity of the initiation of thermal behavior in humans is altered by the extent of cooling/heating of the skin (i.e., heat loss/gain).

Our results thus highlight that in the event of thermal stress, the temperature of the face can modulate the behavioral responses in a bidirectional manner (that is to say both positively on the TS (thermal cooling) and on the comfort thermal (thermal heating).

### T_co_ and Face Cooling

Core temperature is regulated via the control of autonomic and behavioral responses that modulate body heat exchange (Romanovsky, 2007). According to the heat balance equation, when heat gain outweighs heat loss, body heat storage increases; elevating T_co_ and so head temperature. The face and the head are an excellent site for evacuating body heat (Nunneley et al., 1971)and have been investigated as areas for face cooling strategies (Cabanac and Caputa, 1979b). Several studies have shown that head cooling leads to an improvement in TC under the heat stress (Brown and Williams, 1982; Nunneley et al., 1982), but have revealed limited physiological responses, such as T_co_, HR.

In our study, the face was chosen as the site of experimental manipulation due to its small surface area (Brown and Williams, 1982) and because the swimmers wear a silicone swim cap (Hue et al., 2007). During our exercise, swimmers were swimming at the best velocity, and so the metabolic heat production was higher. Consequently, under such conditions, T_co_ rises until heat balance is achieved, as indicated in our study by a plateau at 1,600 m and 2,000 m (Nielsen et al., 1993; Figure 2).

These data indicate that changes in temperature by cold or neutral water in the face are not a requirement for the initiation of thermoregulatory behavior in humans. Face-Cooling and Head-Cooling are particularly effective in improving endurance exercise performance in hot environments, e.g., increasing exercise time to fatigue (Ansley et al., 2008). This is consistent with the emerging evidence that a marked rise in body temperature especially in brain temperature is a major factor in the etiology of fatigue during prolonged exercise in the heat (Nybo et al., 2002). Rather, TS and TC are capable behavioral controllers.

### Heat Rate and Face Cooling

Face-Cooling during passive hyperthermia (Cabanac and Caputa, 1979a), submaximal exercise (Brisson et al., 1989)and maximal exercise are well known to cause bradycardia attributed to a vagal receptor reflex skin stimulated by cold. Borg ratings of perceived exertion are closely related to HR and therefore induced bradycardia could lead to a reduction in perceived exertion (Ansley et al., 2008). However, in our study, cooling of the face did not produce bradycardia during exercise (heat rate was not affected by Face-cooling). This may be due to the phenomenon of acclimatization which may already be present.

In fact, heat acclimatization refers to an increased tolerance to heat during work or exercise under stressful conditions (Nielsen et al., 1993). Acclimatization processes are enhanced in trained subjects, especially in those with high VO_2__max_, and are facilitated by physiological adaptations during repeated exposure. Physiological adaptations of heat acclimatization include an increase in HR related to hyperthermia, stroke volume, sweat rate, and blood plasma volume upon exercise; decrease in HR, T_co_ and mean resting skin temperature (Hue, 2011). Most physiological processes were found to be in place within 8 days in a tropical climate (Voltaire et al., 2002) and in our study our subjects had already been on internship for 8 days. Although most studies have reported the effect of cold water on thermoregulation, swimming in warm water increases the high heat transfer in relation to hyperthermia and the flux to the skin increases in the same way as the running in a hot environment (Holmer and Bergh, 1974). These authors noted an 8 beats.min^–1^ increase in HR from 26^°^C water to 34^°^C water during exercise 20 min of submaximal swimming (approximately 50% of VO_2__max_), most likely related with increase in skin temperature. Thus, the absence of bradycardia due to cooling of the face appears in our study to be linked to swimming in hot water in a tropical climate for acclimatized high-level athletes. It is noted that the speed increases with the cooling of the face. Thus, the absence of bradycardia with cold FC seems linked to more intense exercise. We can therefore think that the subjects of the study, high level athletes, are being acclimatized in tropical climates and so provide better performance day by day. Fatigue accumulated during the training period (already 8 days at the rate of two sessions per day) can also lead to an increase in HR during exercise (Gonzàlez-Alonso et al., 1999).

## Conclusion

To conclude, main result from this study is that psychological factors are modified by cold water splash on face for swimmer, and that may induce an increased velocity and so performance. Face cooling for swimmer performing or training in warm water and tropical climate could enhance session intensity defined as swimming velocity and finally performance.

This study demonstrated that thermal behavior, defined as exercise in water, is influenced by changes in TS and comfort, irrespective of lack of changes in T_co_ and HR. However, both psychological and physiological factors probably play a large role in initiating this behavior (Cheung, 2007; Schlader et al., 2010). This investigation has indicated that changes in temperature are not a requirement for the initiation of thermoregulatory behavior in humans.

### Limitations and Future Research

Our study shows certain limitations: the small number of subjects, and the level of the athletes (highly trained and members of the French team), which did not allow for invasive measurements.

These limits could be exceeded in future research work. A study with a larger number of participants, of different levels of practice, and more complete physiological measurements will help to resolve the questions concerning the absence of physical manifestation during face cooling in swimming in a tropical climate.

## Data Availability Statement

The raw data supporting the conclusions of this article will be made available by the authors, without undue reservation.

## Ethics Statement

The studies involving human participants were reviewed and approved by Ethics Committee of the Training and Research in Sport Science Unit in Guadeloupe (Ministry of Higher Education and Research). The patients/participants provided their written informed consent to participate in this study.

## Author Contributions

All authors contributed to the manuscript redaction, from the plan conception to the review of literature to the corrections.

## Conflict of Interest

The authors declare that the research was conducted in the absence of any commercial or financial relationships that could be construed as a potential conflict of interest. We specify that one of the authors is one of the co-editors. It would be preferable that another editorial team handle this manuscript.
